# Health Tract, No. 96

**Published:** 1862-09

**Authors:** 


### HEALTH TRACT, No. 96.

### SERENITY.

"FRIENDS," commonly known as *Quakers*, as a class live longer than any other persons in the world. The very name of "Quaker" brings up before the mind the personification of equanimity, composure, and quiet dignity. The serene command at once our confidence, our respect, and our love. The brave are serene, and so are the good. In fact, serenity is our highest dignity; it is godlike! And as we should aim to be like Him, in all the qualities possible to man, it is our duty to cultivate serenity, not only because it promotes length of days on earth, and happiness, but does much toward preparation for that after-life whose duration is endless and whose quality is bliss! That serenity of mind is a cultivatable characteristic, is demonstrated by the existence of Friends' Society. Their founders were as other men in birth and habits and propensities; but convictions of certain moral and practical truths came upon them, and they emerged into a new life; they "put off the old man with his deeds," and thereupon framed to themselves a new garb, a "moral dress," which makes them stand out in the world a distinct and an admired people. Peace is a fundamental faith of theirs, and peace is serene. Temperance in all things is another article in their creed, and temperance is serene. Even-handed justice toward all of human kind is the polar-star of their practical faith, and justice is serene. By the practice of these serenities themselves, and by their inculcation upon their children, they have, in half a dozen generations, made it an almost inheritable virtue. While we should cultivate serenity of heart and mind, for the benign influences which it can not fail to have on ourselves and on those with whom we associate, we should be deterred from the neglect of cherishing a quality so divine by keeping in mind the evils which hourly befall those who give a loose rein to the natural man. The great and good Washington is known to have been an extremely irritable man in early life, but he schooled himself to become as calm as a summer's sea in his later years. Our children should be early taught to look calmly at all things, to speak calmly of all things, and judge calmly of all things, and thus avoid those habits of conversational exaggerations, of hasty judgments, of ridiculous praises, and of demoniacal vituperations which so commonly prevail, and which are at once a disgrace to the head and heart of the multitudes who are chargeable in this regard. The want of this self-control, of this calm looking at trouble and at joy, lays us all liable to death without a moment's notice! Mr. P—— died the other day in this city from "some words with a gentleman, which excited him greatly." "He was a man of varied abilities, and had held many high and responsible situations," and might have held them for many years to come had he possessed one other "ability," that of serenity. Mrs. G——, "a lady of high social distinction," on hearing that her nephew had been elected to Parliament, died under the excitement of the gratifying intelligence. Let us then practice ourselves, and teach it to our children, to look at all things, to think of all things, to speak of all things "SERENELY."

